# Genetic Compatibility Determines Endophyte-Grass Combinations

**DOI:** 10.1371/journal.pone.0011395

**Published:** 2010-06-30

**Authors:** Kari Saikkonen, Piippa R. Wäli, Marjo Helander

**Affiliations:** 1 Plant Production Research, MTT Agrifood Research Finland, Jokioinen, Finland; 2 Department of Biology, University of Oulu, Oulu, Finland; 3 Department of Biology, University of Turku, Turku, Finland; Stanford University, United States of America

## Abstract

Even highly mutually beneficial microbial-plant interactions, such as mycorrhizal- and rhizobial-plant exchanges, involve selfishness, cheating and power-struggles between the partners, which depending on prevailing selective pressures, lead to a continuum of interactions from antagonistic to mutualistic. Using manipulated grass-endophyte combinations in a five year common garden experiment, we show that grass genotypes and genetic mismatches constrain genetic combinations between the vertically (via host seeds) transmitted endophytes and the out-crossing host, thereby reducing infections in established grass populations. Infections were lost in both grass tillers and seedlings in F_1_ and F_2_ generations, respectively. Experimental plants were collected as seeds from two different environments, i.e., meadows and nearby riverbanks. Endophyte-related benefits to the host included an increased number of inflorescences, but only in meadow plants and not until the last growing season of the experiment. Our results illustrate the importance of genetic host specificity and trans-generational maternal effects on the genetic structure of a host population, which act as destabilizing forces in endophyte-grass symbioses. We propose that (1) genetic mismatches may act as a buffering mechanism against highly competitive endophyte-grass genotype combinations threatening the biodiversity of grassland communities and (2) these mismatches should be acknowledged, particularly in breeding programmes aimed at harnessing systemic and heritable endophytes to improve the agriculturally valuable characteristics of cultivars.

## Introduction

Mutualistic interactions between microbes and plants are viewed as a ubiquitous cooperation conferring reciprocal benefits to the partners. However, even seemingly highly mutualistic interactions (e.g. between plants, mycorrhizal fungi and/or rhizobia) are inherently unstable, because reciprocal cooperation is based on mutual exploitation and thus costs and benefits are rarely symmetric to the partners [1]–[7]. Consequently, microbial-plant interactions, like any other biological interspecific interaction [1], [2], [8]–[12], involve selfishness, cheating and power-struggles between the partners, thus forming a continuum of interactions from antagonistic to mutualistic [4], with an occasional breakdown in mutualism [12].

The symbiosis between endophytes and grasses is generally considered to be a classic example of microbe-plant mutualism driving grassland communities [13], as well as those food webs subsisting upon them [14], [15]. The close link between endophyte fitness and its host grass is presumed to align the interests of both partners towards a mutually beneficial cooperation [6], [7], [15], [16], a view which seems to be supported by empirical evidence. In this highly integrated symbiosis, hyphae grow intercellularly and asymptomatically throughout the above-ground tissues of the host grass. Through growing into the developing inflorescence and seeds, the fungus is vertically transmitted from maternal plant to offspring. Evolutionary evidence of strictly asexual *Neotyphodium* and sexual *Epichloë* endophytes suggests that such vertical transmission is concomitant with a reduced ability for contagious spreading by asexual or sexual spores and genetic host specificity [16]. Because the fitness and distribution of a fungus largely depends on host fitness [6], any mutualistic cooperation providing a selection advantage to the host plant also benefits the fungus. Conversely, reciprocal benefits from the fungus to the host plant, such as increased growth, resistance to biotic and abiotic stresses and enhanced competitive abilities [15], further support the idea of endophyte-grass mutualism [16].

Nevertheless, in most endophyte-grass interactions partner benefits and symbiotic dependence are asymmetric [6]. Symbiosis is essential for an endophyte because during its systematic growth the fungus subsists entirely on and within the host grass and vertical transmission via host seeds is the primary mode of fungal distribution [16]. By contrast, the symbiotic relationship remains only conditional to the host plant, as plant fitness does not necessarily depend on the fungus [4], [6], [15]. In fact, in some environments symbiosis may even be maladaptive [17], [18]. For example, in endophyte species capable of sexual reproduction, the production of its fruiting body is costly to the host in terms of prevented flowering [19]. Furthermore, in completely asexual endophyte strains, the adaptive value of symbiosis to the host grass appears to vary among fungal strains, being more pronounced in nutrient-rich environments [15], as well as being dependent on plant-plant interactions in grassland communities [13], [20] and trophic interactions in food webs [16], [21], [22], [23]. Accordingly, the infection incidence of grass species and populations appears to be highly variable spatiotemporally [24]–[28], reflecting how fungus and host alike respond to changing selection pressures, either individually or as a phenotypic unit [6].

Here, we use endophyte manipulation trials and a five year common garden experiment to test the importance of genetic compatibility to endophyte-grass symbiosis. Genetic compatibility was examined in three transgenerational phases from the parental plant generation to those of the F_1_ and F_2_ generations; first at the initial encounter of the fungus and the grass, then in the success of the vertical transmission of the fungus to the vegetative propagules (tillers) and offspring of the host grass. The reasoning is that the asymmetric dependence of the endophyte and the host grass may lead to (1) host plant sanctions against less beneficial fungal strains in prevailing selective pressures and (2) the loss of the vertically transmitted fungus, which is continually confronted with new genetic combinations in the out-crossing host population. This is because the endophyte genotype remains unchanged in the plant lineage whilst plant genotypes are blended through recombination over time [6]. This could lead to a genetic mismatch between the fungus and the host, thus destabilizing the symbiosis and constraining the diversity of successful genotype-genotype combinations of the vertically transmitted endophytes and the host grasses.

## Materials and Methods

### Study system

To capture the breadth of variability inherent in grass-endophyte symbioses, as a suitable model we selected wild populations of a native grass species, i.e. red fescue (*Festuca rubra* L.) [29], in subarctic Finland. This species belongs to a large and ubiquitous group of morphologically similar fine leaved *Festuca* species. Because of their high tolerance to a wide range of biotic and abiotic conditions, fescues have been tenacious invaders of terrestrial habitats, colonising every continent on the Earth in greater abundance and distribution than any other group of higher plants [30]. Furthermore, they are also of great agronomic importance in amenity turf. Finnish red fescue populations are infected by the systemic and vertically transmitted *Epichloë festucae* Leuchtm., Schardl & Siegel endophyte [27]. Although a substantial number of native populations are endophyte-free [24], [27], the proportion of infected plants ranges from 4 to 87% in infected populations and is highest in subarctic areas [24], [27]. Furthermore, infection frequencies appear to be habitat-related, being higher in meadows compared to river banks, without genetic differentiation in endophyte populations among the habitats [27]. This suggests that the selection advantage of the symbiosis varies between environments. A single fungal genotype (representing 63.5% of all isolates) dominates the subarctic endophyte populations. The genetic diversity detected appears to be unrelated to either the infection frequency or habitat [27], suggesting that the fungus is predominantly asexual at the edge of its northern distribution range in the subarctic. This supposition is strongly supported by the fact that in our 12 years of intensive fieldwork involving *Festuca rubra* in northernmost Finland, no endophytic sexual fruiting bodies have ever been detected.

### Success of vertical transmission in nature

We collected red fescue (*Festuca rubra*) seeds from 110 wild plants (parental generation) growing in either meadow (six populations; 11, 10, 14, 11, 12 and 10 plants per population) or riverbank (four populations; 10, 10, 10 and 12 plants per population) habitats in subarctic river valleys in northernmost Finland in fall 2000. The infection status of the progenies was first determined by the microscopic examination of stained seeds collected in the field [31], as well as stained leaf sheaths of the established seedlings [32].

### Endophyte manipulations to test genetic host specificity

Our primary intention was to generate F_1_ populations consisting of controlled genotype-genotype combinations of the fungus and the plant (including naturally endophyte-infected (E+) and endophyte-free (E-) controls) to explicitly examine to what extent the phenotypic traits of host-fungal units are explained by fungal associate and genetic combinations between the fungus and the host. The endophyte manipulation trials, involving endophyte removal from E+ seeds by heat treatment, as well as inoculated E- seedlings, also allowed us to examine whether partner specificity or compatibility constrain the diversity of established endophyte symbiosis.

Seeds of 87 maternal plants of the parental generation, producing either strictly E+ or E- progenies, were assigned to the endophyte manipulations. To manipulate interactions between fungal endophytes and grasses, we first eliminated the fungus from some of the E+ seeds by heat treatments (ME-). Using a modification of the method by Williams *et al.* [33], infected seeds were placed in Eppendorf tubes in a water incubator at +54.2°C for 20 minutes. All seeds were germinated and then some of the naturally endophyte-free (E-) seedlings were infected by inoculating hyphae into the plant tissue (ME+) immediately after the emergence of the first seed leaf [34]. The two week old seedlings were potted in sand and grown in the greenhouse. The fungal isolates used in the inoculations were isolated from the same population as each target seedling. The germination rate of untreated seeds was 51%. Although the heat treatment successfully removed the fungus in 99% of the seedlings, the treatment also degreased the germination rate from 51% to 8%. In total, 3397 seeds out of 19831 germinated and 2326 seedlings were subjected to the inoculation treatment. Approximately one third (38%) of the established originally endophyte-free seedlings were successfully inoculated (132 out of 350 seedlings). Inoculation decreased the survival of seedlings from 67% to 30%. Finally, genetic host specificity between the fungus and the host plant was examined with 42 maternal families and 49 fungal isolates. The infection status of the plants was verified in all different phases of the study by growing the fungi out from surface sterilized leaves, and by using a tissue print immunoblot (TPIB) assay [35].

### Common garden experiments

These experiments were designed to examine (1) the success of vertical transmission, and (2) the relative importance of endophyte infection and maternal effects on host grass performance, which can be seen as potentially adaptive responses to the maternal environments [36]. Here, naturally and artificially infected and endophyte-free seedlings, i.e. E+, ME+, E- and ME-, respectively were transplanted from the greenhouse to a common garden at Ruissalo Botanical Garden, Turku, in 2003. In total, 679 seedlings comprising 134 and 72 E+, 39 and 35 ME+, 184 and 139 E- and 35 and 41 ME- of 75 maternal plants originating from meadows (48 plants) or river banks (27 plants), respectively, were randomly assigned within 19 blocks and planted in sand.

#### Success of vertical transmission

To determine the success of vertical transmission in vegetative tillers (F_1_ generation), four tillers were gently detached from 10 E+ plants (maternal environment: 6 and 4 from meadows and river banks, respectively) and from 10 ME+ plants (maternal environment: 4 and 6 from meadows and river banks, respectively) growing in the common garden in 2007. The tillers were then transplanted separately into 7.5×7.5 cm pots containing sand and grown in the greenhouse for two months, after which their infection status was determined from all or at least 10 of the tillers, depending on their size.

To determine any transgenerational reduction in infection in the sexually produced F_2_ generation, we collected all seeds of experimental plants in August 2004, germinated and planted them, and determined the infection status of the established seedlings.

#### Importance of endophyte symbiosis and maternal effects

To examine relative importance of endophyte symbiosis and maternal effects, plant performance was examined as biomass in 2004, including the number of inflorescences produced each year (2004–2008).

#### Statistical analyses

We used Chi-square analyses (χ^2^ –test) to test the effects of endophyte manipulation (natural or introduced) on loss of infection during vegetative growth and seed production, as well as any differences in endophyte inoculation success among originally E- seed families and endophyte isolates. In total, 23 originally E- seed families and 33 endophyte isolates producing at least five established seedlings were included into the statistical analyses to meet the requirements of the χ^2^ -test [37]. The effects of the original habitat (meadow or riverbank) and endophyte status on one year biomass production in the common garden was analysed with the GENMOD procedure, using gamma distribution and power (−1) as a link function. We examined inflorescence production in common garden grasses in 2004–2008 using two methods: firstly, with a repeated measures model and then separately for each year with GENMOD (negative binomial distribution and log-link function). The variables original habitat, endophyte status and their interaction were used as fixed factors in the statistical models. Analyses were done with the SAS software package version 9.1 (SAS Institute, Cary, NC, USA).

## Results

Vertical transmission of the fungus is imperfect in nature. Of the 110 plants collected in the field, 58 hosted the endophytic fungus, of which 23 produced both endophyte-infected and endophyte-free seedlings in the F_1_ progeny. This demonstrates that the fungal infection was lost in some seedlings of the offspring in 40% of the endophyte-infected maternal families in nature.

**Figure 1:**
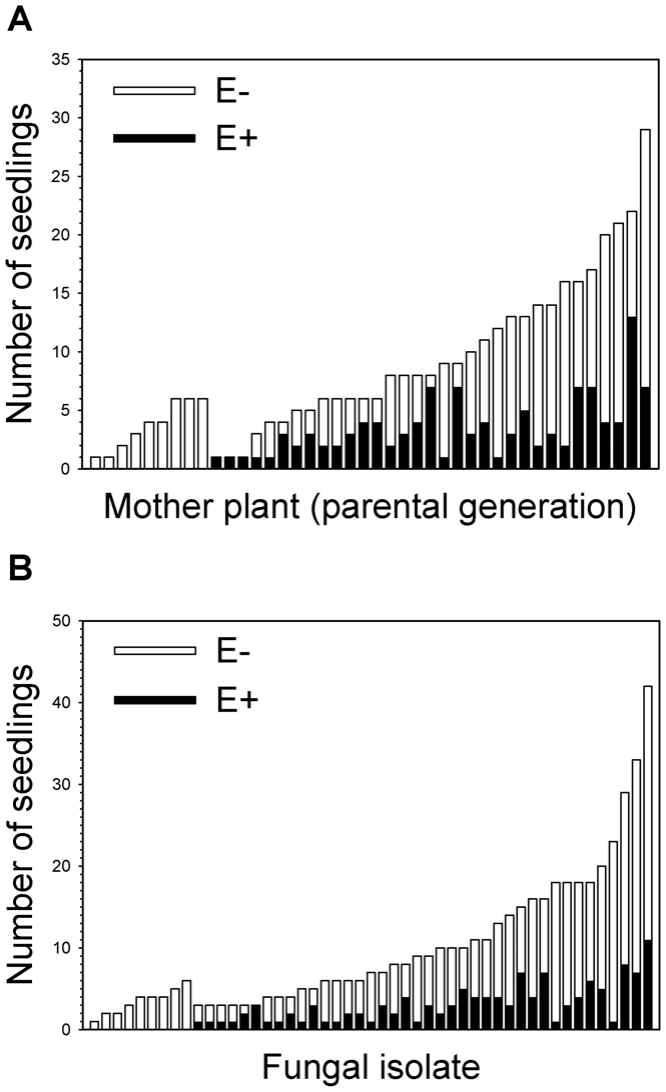
Success of manipulation trials. Inoculation success of fungal endophyte isolates in manipulation trials with 42 naturally endophyte-free maternal families of *Festuca rubra* (A) and 49 *Epichloë festucae* endophyte isolates (B) isolated from the same populations. Endophyte-infected (E+) and endophyte-free (E-) seedlings are shown as black and white bars.

Endophyte manipulation trials suggest that the genetic compatibility between the fungus and the host drives the symbiosis. One fifth of the F_1_ progenies did not accept the endophyte at all, inoculation success varying from 0 to 88% among the host plant progenies (χ^2^ = 42.25, df = 22, p = 0.008) (Fig. 1 a). Similarly, inoculation attempts were unsuccessful with nine fungal isolates, and inoculation success among the 49 fungal isolates varied from 0–100% (Fig. 1 b). However, the difference in infectivity among endophyte isolates remained statistically insignificant (χ^2^ = 35.40, df = 32, p = 0.311) when only the cases of at least five established seedlings were included in the analyses to meet the requirements of the χ^2^ -test [37]. Nevertheless, the inoculation success varied from 0–60% in these 33 cases (Fig. 1 b). These results suggest that a realized assortment of the endophyte-grass symbioses is primarily determined by the host grass genotype and can be partly confined to well-matched genotype-genotype combinations of the endophytes and the host grasses.

**Figure 2:**
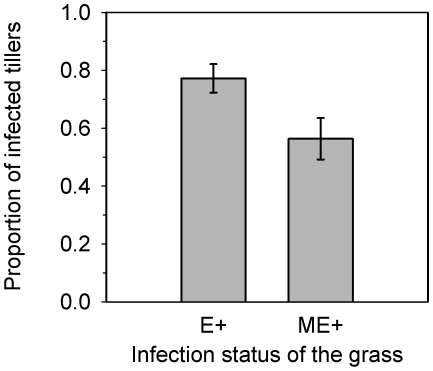
Loss of infection in vegetative grass tillers. Proportion (%) of *Epichloë festucae* endophyte-infected tillers of naturally (E+) and manipulatively (ME+) endophyte-infected *Festuca rubra* plants in F_1_ generation. The means are calculated for each mother plant (n = 10). Error bars show S.E.

**Figure 3:**
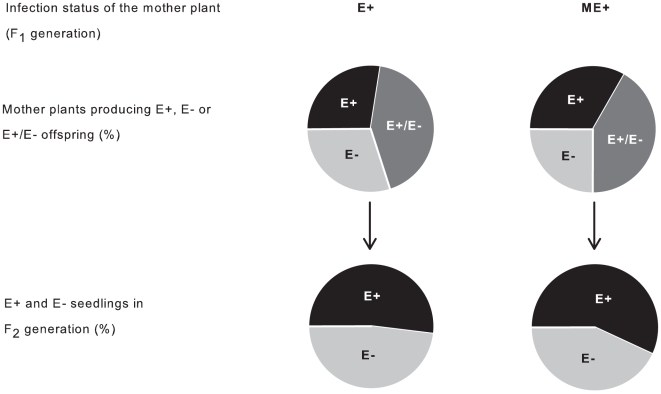
Loss of infection in sexually produced seedlings. Proportional (%) loss of endophyte infection in *Festuca rubra* seedlings (F_2_ generation). Of the 47 endophyte-infected (E+) mother plants (F_1_ generation), 14 produced offspring of exclusively endophyte-infected seedlings (total = 20), 13 produced offspring of exclusively endophyte-free seedlings (total = 19), while 20 mother plants produced progenies consisting of both E+ and E- seedlings (44 and 37, respectively) in the F_2_ generation. Similarly, three and four out of 12 manipulatively endophyte-infected (ME+) mother plants produced offspring of exclusively endophyte-infected (total = 3) or –free (total = 4) seedlings, and five of the mother plants produced progenies consisting of both E+ and E- seedlings (10 and 13, respectively) in the F_2_ generation. In total, E+ mother plants produced 63 and 57, and ME+ mother plants, 21 and 16 established E+ and E- seedlings, respectively.

In the common garden and greenhouse experiments, the loss of infection was pronounced and appears to be partly attributable to the genetic mismatch between the fungus and the host. Firstly, the infection was lost in 33% of vegetative grass tillers (Fig. 2) and 46% of sexually produced seedlings (Fig. 3), which demonstrates that the loss of infection can be substantially more frequent than has been commonly assumed in previous literature [4], [6], [16], [24], [27] and higher in seeds produced by outcrossing compared to clonally produced plants. Secondly, consistent with the hypothesis of genetic mismatch, fungal growth in vegetative tillers was 27% lower in ME+ maternal families compared to E+ families (Fig. 2; χ^2^ = 12.27, *df* = 1, *P*<0.0005). Meanwhile sexually produced offspring of E+ and ME+ plants comprised of only E-, a mixture of both E+ and E- and only E+ progenies in similar proportions (Fig. 3; χ^2^ = 0.1835, *df* = 2, *P*<0.9123). The greater infection instability in vegetative tillers in ME+ plants can be expected because the novel fungal-grass genotype combinations were not tested and selected for by natural selection. Instead, according to the hypothesis, transgenerational instability should be equal in ME+ and E+ plants in common garden where cross-pollination freely occurs between the experimental plants and nearby wild grasses, thereby increasing genetic mismatch between the fungus and the host. This creates an unfit endophyte-grass genotype combination and destabilizes the symbiosis.

**Figure 4:**
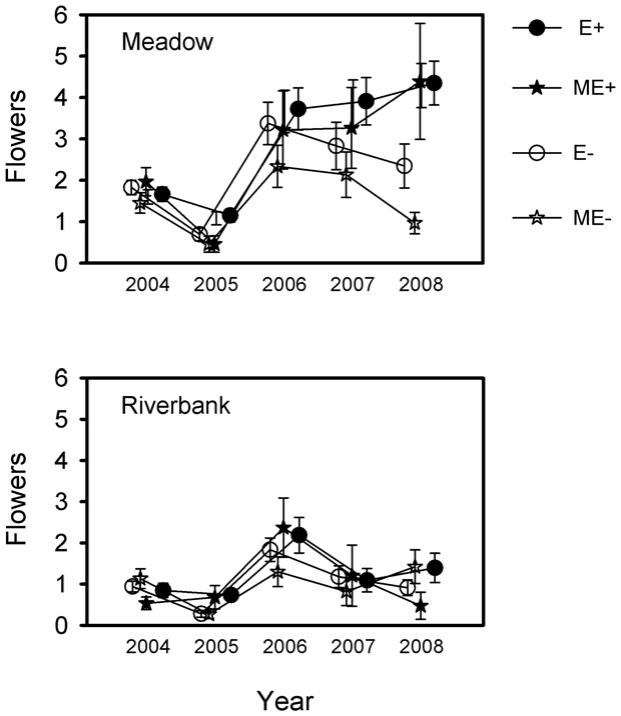
Inflorescence production of plants. The number of inflorescences of infected (E+), manipulatively infected (ME+), endophyte-free (E-) and manipulatively endophyte-free (ME-) *Festuca rubra* plants (F_1_ generation) originating from meadow and river bank habitats (parental generation) growing in a common garden between 2004–2008 (yearly means ± S.E.).

In 2004, the biomass of common garden grasses was affected by habitat (χ^2^ = 12.23, *df* = 1, *P*<0.0005), but not by endophyte infection (χ^2^ = 1.61, *df* = 3, *P*<0.6571) or their interaction (χ^2^ = 0.74, *df* = 3, *P*<0.8646). Meadow grasses showed a slightly higher biomass (mean±SE: 0.465±0.012 g) than river bank grasses (mean±SE: 0.402±0.013 g). The removal of the above-ground biomass of plants decreased number of inflorescences in the following year 2005 (Fig. 4).

There was a three-way interaction between year, habitat (meadow or riverbank) and endophyte status in inflorescence production of common garden red fescues (Table 1). In each study year, meadow grasses produced more inflorescences than those from the river bank (Table 2, Fig. 4). Endophyte-infected grasses from both habitats produced more inflorescences in 2005 compared to uninfected plants. In 2008, endophyte-infected grasses from meadows again produced more inflorescences than uninfected grasses. By comparison, endophyte-removed (ME-) grasses produced far fewer inflorescences compared to others (Table 2, Fig. 4).

**Table 1:** Repeated measures analysis for inflorescence production of *Festuca rubra* grasses in a common garden.

Explanatory variable	df	χ^2^	p
Habitat (H)	1	26.00	<.0001
Endophyte status (E)	3	12.22	0.0067
H*E	3	0.98	0.8052
Time (T)	4	71.06	<.0001
E*T	12	22.25	0.0348
H*T	4	11.93	0.0179
H*E*T	12	25.22	0.0138

**Table 2:** Effects of original habitat and endophyte status on inflorescence production of *Festuca rubra* grasses grown in a common garden in different years.

		2004		2005		2006		2007		2008	
Exp. Var.	df	χ^2^	p	χ^2^	p	χ^2^	p	χ^2^	p	χ^2^	p
Habitat (H)	1	24.1	<0.001	3.3	0.068	10.6	0.001	21.2	<0.001	21.4	<0.001
Endo (E)	3	1.7	0.641	15.4	0.002	5.5	0.138	2.9	0.416	10.8	0.013
H*E	3	2.9	0.401	5.3	0.153	0.7	0.880	1.3	0.719	12.5	0.006

Abbreviations: Exp.Var. = explanatory variable, Endo = Endophyte status.

## Discussion

Our results demonstrate that genetic mismatches, maternal effects and loss of infections occur commonly in endophyte-grass interactions and may partly explain those differences detected in infection frequencies and genetic structures among natural grass populations [6], [36]. For example, seeds produced by outcrossing should have a high frequency of mismatches between the fungus and the grass and thus pioneer grass populations having a large portion of newly established individuals should have lower infection frequencies than older populations. The higher frequencies of endophyte-infected grasses detected in meadows compared to sandy river banks support this view [27]. Meadows are more stable and fertile environments, whose grass populations are older and well established by clonal spread. By contrast, riverbank populations suffer almost annual disturbance due to spring flooding [27], [38]. Furthermore, our results indicate that the maternal environment may strongly affect inflorescence production of the grasses with a time lag and thereby the dispersal and competitive ability of endophyte-infected plants. As predicted by the geographic mosaic theory of co-evolution [11], endophyte–plant interactions appear to project to the hot- and cold-spots of selective pressures in an ecosystem. Accordingly, endophytes may provide selective advantages to the host in some environments [27], [39], which are occupied by the locally most fit fungus–plant genotype combinations within a population. Consequently, in nature the established endophyte-host combinations clearly represent only a fraction of the available genetic variation in the populations.

The fragility of endophyte infections detected in this work questions the strict mutualistic nature of endophyte-grass symbiosis and suggests that the high frequencies of endophyte-infected plants in subarctic red fescue populations may only persist if the selective advantage of endophyte infection to the host plant is high. Because the endophyte may increase its distribution and fitness primarily by increasing the plant's allocation to female functions, we counted the number of inflorescences in the common garden experiment over a five year period. On average 44% of plants produced inflorescences each year. Endophyte infection increased inflorescence production of grasses in some years regardless of the manipulation status of the infection. This difference in inflorescence production between endophyte-infected and endophyte-free grasses was more pronounced and frequent in meadow grasses (Fig. 4). This may partially explain the higher infection frequencies detected in meadows compared to river banks [27]. Interestingly, the production of inflorescences in ME- plants declined towards the end of the experiment, suggesting that the loss of the endophyte after a long co-evolutionary relationship may be disadvantageous to host plant fitness when long-term reproductive success is taken into account. These results emphasize the importance of both long-term experiments and demographic records from natural populations in ecological and evolutionary studies.

Our findings have theoretical and methodological implications, with potential economic value in agronomy as well. Firstly, these results illustrate the importance of genetic incompatibility in out-crossing grass populations and maternal effects as destabilizing forces in endophyte-grass symbioses. These forces may act as buffering mechanisms against competitive endophyte-grass genotype combinations, potentially dominating the populations and grassland communities. Secondly, genetically determined resistance to endophytic fungi and genetic mismatch that constrains combinations of fungi and host grass, should be acknowledged in breeding programmes aimed at improving agriculturally valuable characteristics of cultivars such as higher yield and resistance to herbivores and pathogens.
